# Caregiver’s perspectives on the Central Nervous System infection illness trajectory among older persons with dementia in Northern Uganda—a qualitative community-based study

**DOI:** 10.1186/s12877-022-03499-1

**Published:** 2022-10-27

**Authors:** Deo Benyumiza, Edward Kumakech, Jastine Gutu, Jude Banihani, Joshua Mandap, Zohray M. Talib, Edith K. Wakida, Samuel Maling, Celestino Obua

**Affiliations:** 1 Department of Nursing and Midwifery, Faculty of Health Science, Lira University, P.O. Box 1035, Lira City, Uganda; 2 Office of the Health Professional Education Partnership Initiative – Transforming Ugandan Institution’s Training Against HIV/AIDS (HEPI-TUITAH) Program Administration, Faculty of Health Science, Lira University, Lira, Uganda; 3 Department of Medicine, California University of Science and Medicine, San Bernadinio, USA; 4 Office of Research Administration, Mbarara University of Science and Technology, P.O. BOX 1014, Mbarara, Uganda; 5 Department of Psychiatry, Faculty of Medicine, Mbarara University of Science and Technology, Mbarara, Uganda; 6 Office of the Vice Chancellor, Mbarara University of Science and Technology, Mbarara, Uganda

**Keywords:** Dementia, Caregivers, Older persons, CNS infections, Northern Uganda

## Abstract

**Background:**

Few studies have explored the Central Nervous System (CNS) infection illness trajectory among older persons with dementia in sub-Saharan African (SSA) settings. This study explored the Caregiver’s perspectives on the Central Nervous System infection illness trajectory among the older persons with dementia in Northern Uganda.

**Methods:**

This was a qualitative study conducted in Lira District northern Uganda in March 2022 amongst purposively selected 20 caregivers of the older persons aged 50 + years with a positive history of CNS infection and later life dementia. Data were collected using an in-depth interview guide. Audio recordings and field notes of the interviews were undertaken. The interviews generated data on the CNS infection illness trajectory from onset to the current demented state of the older persons. The audio-recorded interviews were transcribed verbatim before manual reflective thematic analysis.

**Results:**

Older persons with a positive history of CNS infection illness and later life dementia in rural northern Uganda presented with symptoms of early life CNS infection illness ranging from neck pain, back pain, chronic headache, and fatigue. There were also manifestations of comorbidities particularly road traffic accidents involving traumatic injury to the head, neck, and spine, high blood pressure, chronic headache, and or their medications in the older person’s trajectory to later life dementia. A plurality of healthcare which included both formal and informal healthcare medicines was sought and utilized for the treatment and care of the CNS infection illness and dementia by the older persons amidst improper diagnosis and mismanagement.

**Conclusions and recommendations:**

Older persons with early-life CNS infections illness and later-life dementia were reported to present with symptoms including neck pain, back pain, chronic headache, high blood pressure, and fatigue. The reported symptoms of CNS infection illness may be intertwined with co-morbidities particularly traumatic injury involving the head, neck, and spine, high blood pressure, and chronic headache. Healthcare professionals should integrate routine screening of older persons for the history of CNS infections, chronic headache, high blood pressure, trauma to the head, neck, and spine, and dementia and early treatment.

## Study background

Dementia is a syndrome in which there is deterioration in cognitive function beyond what might be expected from the usual consequences of biological aging [1]. More than 55 million people are living with dementia worldwide and nearly 10 million new cases occur every year [1]. Over 60% of the persons with dementia live in low-and middle-income countries and this proportion is expected to rise to 78 million in 2030 and 139 million in 2050 due to increasing population of older persons worldwide [1, 2]. In sub-Sahara Africa (SSA) by 2017, over 2.13 million people were living with dementia and this was projected to double every 20 years, increasing to 3.48 million and 7.62 million by 2030 and 2050 respectively [3]. In Uganda by 2017, Alzheimer’s Disease and related dementia (ADRD) deaths were approximated at 2,376 (0.925%) of the total deaths [4].

Central Nervous System (CNS) infections have for decades been epidemiologically and sometimes mechanistically associated with dementia among older persons globally [5–11]. Infections involving the brain and spinal cord are associated with substantial morbidity, mortality, or long-term sequelae that may have catastrophic implications for the quality of life of affected individuals [12]. Microorganisms that cause CNS infections include a wide range of bacteria, mycobacteria, yeasts, fungi, viruses, spirochetes (e.g., neurosyphilis), and parasites (e.g., cerebral malaria and strongyloidiasis) [6, 13–16]. The CNS infections further fall into three broad categories i.e. meningitis, encephalitis, and abscesses [6, 12].

The CNS infection may be spread from blood-borne infections, infections at sites adjacent or contiguous to the CNS such as the mastoid, sinuses or middle ear or from primary infections at more remote anatomic sites such as lungs, and heart, skin, gastrointestinal tract, or the kidneys [6, 12]. Clinical features of the various CNS infections can be non-specific or characterized by distinct, recognizable clinical syndromes [12]. Older persons are at higher risk of suffering from CNS infections due to the weakening immune system with the advancing age and longer exposure to the environmental factors which increases the chances of developing dementia following CNS infections among older persons [17].

In Uganda, there is an increasing population of older persons [18, 19] who require special attention to reduce the burden of dementia [20]. Despite this trend, there are limited guidelines on the management of Alzheimer’s Disease and Related Dementia (ADRD) in Uganda. Resources from the World Health Organization (WHO) such as *Towards a Dementia Plan – A WHO guide*, *guidelines on risk reduction of cognitive decline and dementia*, m*dementia handbook*, *WHO mental health gap action program (mhGAP)* and *Towards a dementia-inclusive society – WHO toolkit for dementia friendly initiatives* have insufficiently addressed CNS infection’s considerations in dementia risk reduction, prevention and care.

Worse still, fewer previous studies investigated the CNS infection illness trajectory among older persons with probable or possible, dementia especially from sub-Saharan African (SSA) context. The few available previous studies in the SSA focused on the prevalence and risk factors for HIV-associated neurocognitive disorders including HIV-associated dementia [6, 21–23].

An CNS infection illness trajectory is defined as the occurrence of factors, events, signs, and or symptoms with or without causative—outcome roles in the past and present life history of the older persons with possible or probable dementia. Investigations into CNS infection illness trajectory among older persons with probable or possible dementia are critical in risk reduction and prevention of severe forms of dementia through early screening, and diagnosis of persons with signs and symptoms of CNS infection illness.

Lira district located in northern Uganda has an increasing population of older persons [19]. However, screening for CNS infection illness and probable or possible dementia among older persons is not a routine practice among healthcare providers in Uganda due to limited capacity [24] and most likely so in other sub-Saharan African countries. No routine care for dementia implies that the older persons miss out on early diagnosis, risk reduction interventions and the care for probable or possible dementia. The aforementioned problems along with limited research attributed to the knowledge gap on the common CNS infection illness symptoms to screen for amongst older persons at risk for dementia presenting to healthcare, which this study attempted to address. The research results can inform the development of policies and guidelines on CNS infection illness screening, risk reduction interventions, treatment and care for older persons at risk of dementia.

This study therefore explored CNS infection illness symptoms among older persons aged ≥ 50 years in Lira District northern Uganda with positive history of CNS infection illness who screened positive for probable or possible dementia with the BCSD. Notably, the definition of older persons from a seemingly early age of 50 years was used in a sister quantitative study upon which this qualitative study was piggybacked meant for screening possible or probably dementia including early onset cases as early as 50 years.

## Methods

### Study design and setting

This qualitative inquiry was prompted by a preceding quantitative survey which found a significant association between possible or probable dementia and positive history of CNS infection illness among older persons aged 50 + years in northern Uganda. We conducted a descriptive qualitative study [25]. The descriptive design allowed for elaborate description of the CNS infection illness symptoms in their natural states from the respondent’s points of views. The study was conducted in 20 villages in Lira District Northern Uganda. The villages were rural and suburb of a city in human settlement context. Lira District is approximately 337 km by road, north of Kampala the capital city of Uganda.

### Population and sample size

The study was conducted among the key informants who were the caregivers of the older persons aged ≥ 50 years with probable or possible dementia [from the Brief Cognitive Score for Dementia scale (BCSD) screening] and a positive history of CNS infection illness. Caregivers are defined as the spouse or relative providing general home-based care for the older persons with dementia by providing support in form of house cleaning, cooking, bathing, washing, shopping, grooming, walking support and or accompaniment to the healthcare facilities among other activities of daily living. We used the maximum variation purposive sampling method to recruited 20 participants who lived the experience of being caregivers of the older persons with the phenomena under scrutiny (i.e. CNS infection illness symptoms trajectory among their relatives or partners with probable or possible dementia). This being a qualitative key informant study the variations considered during the purposive sampling of the key informants were the caregiver’s age, sex, educational level, and relationship with the older person with possible or probable dementia under their care rather than the CNS infection illness characteristics among the older persons with dementia (Table 1). Only one main caregiver was selected per older person with dementia. The 20 caregivers therefore represented 20 older persons with possible or probable dementia and positive history of CNS infection illness. The field guides in this case the village health teams (community health workers) and other family members living in the older person’s homestead helped in identifying and confirming the main caregivers for each older person with possible or probable dementia and positive history of CNS infection illness. Caregivers of older persons with probable or possible dementia but negative histories of CNS infection illness were excluded from the sample. Similarly, no CNS infection illness data were collected from the older persons with possible or probable dementia in this qualitative key informant study because the screening tool (BCSD) does not categorize cognitive functions into mild, moderate nor severe dysfunctions but rather into unlikely dementia, possible or probable dementia. The principle of data saturation, a point at which further sample did not add any new insights was used to determine the sample size of 20 participants to develop robust understanding of the phenomenon [26].

**Table 1:** Description of the socio-demographic characteristics of the study participants who were caregivers of the older persons with positive history of CNS infection illness and later life dementia in Lira district northern Uganda

Participants	Age in years	Gender	Level of education	Relationship with the patient
P 1	68	Female	Primary education	wife
P 2	59	Female	Lower secondary	sister
P 3	50	Male	Primary education	Husband
P 4	78	Female	No formal education	sister
P 5	66	Male	Primary education	Husband
P 6	58	Male	Lower secondary	Husband
P 7	65	Female	Lower secondary	Sister
P 8	75	Female	Primary education	Grand-child
P 9	55	Female	No formal education	Sister
P 10	57	Male	Primary education	Husband
P 11	53	male	Secondary education	Husband
P 12	64	Male	Primary education	Husband
P 13	69	Female	No formal education	Son
P 14	56	Female	No formal education	Wife
P 15	57	Female	Primary education	Daughter
P 16	71	Male	Tertiary	Son
P 17	80	female	No formal education	Sister
P 18	62	Female	Primary education	wife
P 19	57	male	Secondary education	Husband
P 20	84	female	No formal education	Wife

This qualitative study was piggybacked on the quantitative household survey which investigated for the association between CNS infection illness and dementia among older persons in the study setting and helped to identify participants with both positive history of CNS infection illness using a CNS infection questionnaire and probable or possible dementia using a Brief Cognitive Score for Dementia scale. From the preceding quantitative survey analysis, a total 49 older persons met the criteria of having possible or probable dementia and positive history of CNS infection illness and it provided the sampling frame for the selection of key informants for the qualitative research. The preceding quantitative household survey of older persons in northern Uganda for prevalence of dementia and its association with CNS infection illness was conducted in March 2022. It involved a sample of 434 older persons aged 50 + years in Lira district northern Uganda. The quantitative household survey found a high prevalence of possible or probable dementia (23%) among older persons aged 50 + years in northern Uganda. It also found a significant association between possible or probable dementia and positive history of CNS infection illness among older persons aged 50 + years in northern Uganda at both bivariate and multi-multivariate analysis. The quantitative survey paper is under peer review for journal publication.

### Participants’ recruitment procedures

The selection of the study setting for this descriptive qualitative study was informed by a preceding quantitative household survey of older persons in the aforementioned 20 villages in Lira district northern Uganda for dementia among older persons and its association with CNS infection ilness. Older persons who were found to have a positive history of the CNS infection illness and dementia from the quantitative household survey were eligible for this qualitative study. The Community health extension workers (CHEWs) also known as Village Health Teams (VHTs) who enlisted the households with older persons aged ≥ 50 years for the quantitative household survey also helped to identify and locate the residential addresses of the older persons with positive history of the CNS infection illness and later life dementia.

The VHTs are community health volunteers who maintain registers of all residents including older persons in their respective villages [27]. The VHTs are lay members of the villages with basic primary or secondary school education level who are selected by their respective village members to assist in the distribution of health commodities such as posters, contraceptives, mosquito bednets and also mobilize fellow village members for health events such as health education talks, immunization, disease tests etc. by health workers at the community health outreach posts. After selection, the VHTs receive a 3-months in-person training by the Uganda Ministry of Health and partners on how to maintain the village health register, mobilize fellow village members for health events and distribute health commodities such as posters, contraceptive pills, condoms, mosquito bed nets etc. The village register is used for the mobilization of targeted residents for health education, counseling, screening or testing for diseases. The VHTs also use the register for targeted distribution of health communities such as contraceptive methods and linkages of patients from the community to health facilities for clinical healthcare. During the study, the VHT assisted in enlisting of households with older persons aged 50 + years, field guiding to the households and identifying the main caregivers of the older persons with possible or probable dementia from among the household members.

### Data collection method, tools and procedures

We conducted the face-to-face in-depth interview using an interview guide. The interview guide comprised of open-ended questions to elicit the participants account of the CNS infection illness trajectory among older persons with probable or possible dementia who were their care receivers. The interview guide was developed by the research team members informed by literature. The items in the guide were pre-tested among caregivers of the older persons who were not included in the study and improvements were made accordingly. The interview guide had three sections. The first section comprised of the socio-demographic characteristics of the study participants. The second section comprised of items for confirming the prior CNS infection illness diagnosis or the history of CNS infection illness, the characteristics and hence the types of the CNS infection illness and dementia among the older persons. The third and last section of the guide comprised of items on the CNS infection illness trajectory from onset to the current demented state of the older persons. The Research Assistants with nursing and midwifery educational background who were trained on the research protocol asked the questions in an open-ended format to elicit responses and conversations for each section of the interview guide. The interviews were conducted in the local language Luo. Interviews were conducted in private spaces within the participants’ homes. The interviews lasted for 30 to 60 min. Interviews were audio recorded. Field notes were also taken by the Research Assistants on the body language and unique circumstances around the participant at the time of data collection.

### Data management and analysis

The audio recorded data were transcribed verbatim by the Data Clerk. The transcripts were verified against the source audio recordings by a research team member to clarify any unclear data before permitting the Data Clerk to conduct the next transcription. Verbatim transcripts in the local language (Luo) were translated to English language before analysis. Two research team members independently coded the data and discrepancies in codes were reconciled through consultation with other research team members. We conducted a manual reflexive thematic analysis of data as they manifest from the participants in accordance with the six steps described by Braun and Clarke [28]. Findings were presented as contextual description of the CNS infection illness symptoms trajectory among the older persons with possible or probable dementia while providing supporting direct quotations from the interviews.

### Scientific rigor and trustworthiness

The four principles of credibility, dependability, confirmability, and transferability of qualitative research were upheld to assure and maintain rigor and trustworthiness in the study. Credibility was assured by restating and summarizing points during the interviews and also by taking field notes from the interviews in addition to the audio recording [29]. Dependability and confirmability were assured by audio-recording of the interviews against which the correctness of the verbatim transcripts, findings, and interpretations were validated [29]. Provision of many direct quotations (participant’s voices) to support emergent themes also assures dependability and confirmability of the data. Finally, transferability was assured by providing a thorough description of the study setting and data collection methods including the underlying assumption that CNS infection illnesses could be involved in older persons with dementia [29]. We also provided a detailed description of the study area, setting, participants and methods to assure transferability of the study findings.

## Results

We approached the 20 purposively selected caregivers of the older persons aged ≥ 50 years to provide informed consent and all of them consented and participated in the in-depth interviews. The response rate was therefore 100%.

### Socio-demographic characteristics of study participants

The socio-demographic characteristics of the 20 caregivers of the older persons aged ≥ 50 years with a positive history of CNS infection illness and later life dementia in Lira District northern Uganda who participated in the study is shown in Table 1. The age of the participants ranged from 50 to 84 years. The majority of the participants were subsistence farmers (80%), females (60%), married (45%) and 40% had attained primary level of education.

### Theme 1: presenting early symptoms of illnesses

Some symptoms of the CNS infection illnesses surfaced at an earlier time or age of the demented person’s life. These early symptoms in terms of their earlier time of manifestation in the demented person’s life are therefore put together under the theme of presenting early symptoms of the CNS infection illness although they varied considerably in terms of the actual time of manifestations in the demented person’s life ranging from childhood, adolescence, youthful age, middle age though a few years or months ago.

#### Complaining of neck pain and back pain

We explored the initially presenting symptoms of CNS infection illness among the older persons prior to being screened positive for probable or possible dementia. Many of the key informants (who were caregivers) narrated that the older persons with probable or possible dementia were always complaining of pain around the neck and the back. From these, it slowly progressed to memory loss and inability to carry on with daily activity. The experiences of neck and back pain was typified by a response from one of the study participants described below.

*“The problem she had especially now that you have come to make a follow up, her main problem she used to complain of pain. This complain went on until a point that it become worse to the extent that she started forgetting a lot of things. Her major complaint was that the pain would start from the lower limbs, radiates to the waist region then to the back through her spinal cord”* (Participant 4, 78 years, female, Sister)

#### Getting fatigue

Some key informants reported that the older persons with probable or possible dementia also complained of fatigue. This would also interfere with their ability to carry on daily activities and raised the suspicion that something was not right with their health. Some key informants went on to say that the feeling fatigue increased stress levels among the older persons. They thought the fatigue was the part of the reason as to why their relative with probable or possible dementia suffered from memory loss. One of the participants had the below to say.

*“it all started one day as she was here on the compound winnowing sorghum when she realized her energy was getting low and she decided to go and take some rest, when I went inside to check on her after sometime I found she was struggling with breath, I tried calling her she could not respond to me I then ran to my elder brother who came and picked her and she was taken to the hospital. And another thing I know is she subjects herself into too much worry”* (Participant 7, 65 years, female, Sister)

#### Having chronic headache

Some key informants pointed out chronic headaches as one of the complaints that were always raised by the older persons who were screened positive for probable or possible dementia. Chronic headache typically is due to the inflammation of the immune system secondary to the CNS infection. This continuous headache without concise remedy could have led to development of the dementia among the older persons. This was described by one participant below.

“*My relative always complained of having too much headache and sudden feeling of being very tired, it was then at that point we took her to the hospital and she was confirmed to be having illness which was said had started to affect her brain”* (participant 9, 55 years, female, Sister)

### Theme 2: manifesting co-morbidities and or their medications

The study also investigated prior conditions amongst the older persons with a positive history of CNS infection and dementia. These prior disease conditions or illnesses that co-existed in the demented persons with positive history of CNS infection illnesses were defined under the theme of manifesting co-morbidities and or their medications. The findings are presented below.

#### Suffering road traffic accidents

Some of the key informants revealed that the older persons have been victims of road traffic accidents that led to shock and paralysis for some time. And from the accident they started experiencing signs of forgetfulness and memory loss. Traumatic tissue injury from road traffic accidents are often entry point for some of the pathogenic microbes that infect the central nervous system as many of the participants also reported that from the time their relative were involved in two rows of the accidents they started suffering from episode of CNS infection illness. This was typified by one of participants below.

*“Her condition started after she had suffered an accident and hit her head on the ground. suffered two accidents in a row and after the accidents, hit her head on the ground, she started experiencing memory loss, back and body pain, victim of Goiter, shock, suffered a stroke. Half of her body was completely paralyzed for some time, and she kept suffering from the brain infection which made her forget a lot of things. Keeps getting malaria infections”* (participant 3, 50 years, male, Husband)

#### Having episodes of forgetfulness after taking drugs for high blood pressure

Other key informants reported that their relatives (the older persons) started experiencing episodes of forgetfulness following treatment for high blood pressure. They related the forgetfulness to the side effects of the medications they gave their relatives (the older persons) as treatment for high blood pressure. The thiazide diuretic and a calcium channel blocker (nifedipine) the two widely available and commonly prescribed antihypertensive drugs in Ugandan public health facilities were implicated by the key informants. This was exemplified by one of the key informant’s comment below.

*“What I can tell you according to my understanding about how this problem begun is she was taken to the hospital and was diagnosed with pressure and she was given medication to help control the condition, it was then when she started on medication when we realized she started having episodes of forgetfulness but this was not happening before she was put on medication. And so people started telling us it could be the side effects of the medication that she is taking to control pressure which is causing her to be acting that way”* (participant 6, 58 years, male, Husband)

#### Complaining of chronic headache

Some key informants also pointed out chronic headache as one of the complaints that were always raised by these older persons indicated for probable dementia. This could be due to the inflammatory response of the immune system to the CNS infection that triggered the headache. This continuous experiencing of headache with no concise remedy for the infection-related CNS illness could have led to development of signs dementia among the older persons aged ≥ 50 years. This is quoted by one participant.

“*My relative always complained of having too much headache and sudden feeling of being very tired, it was then at that point we took her to the hospital and she was confirmed to be having illness which was said had started to affect her brain”* (participant 9, 55 years, female, Sister)

#### Having episodes of forgetfulness after being diagnosed for high blood pressure

Many of the key informants reported of their relatives (the older persons) being ever diagnosed with high blood pressure in addition to the diagnosis of CNS infection and dementia. The participants believed having hypertension weakened their immune system and made them vulnerable to CNS infections and developed dementia. This was exemplified by a narrative from one of the study participants below.

*“She looks young though already aged than I am, my sister was once diagnosed with an underlying condition of high blood pressure, which I hope has made her weak and very vulnerable to CNS infections and forgetting things”* (participant 2, 59 years, female, Sister)

### Theme 3: seeking and receiving of series of treatment and care without thorough checkup

The study explored the diagnosis, treatment and care sought and given to the older persons when experiencing the signs and symptoms of illness before the diagnosis of CNS infections and the positive screening test for dementia. Therefore, this theme of seeking and receiving of series of treatment and care without thorough checkup for CNS illnesses consolidates all the missed diagnosis, unsuccessful treatment and care sought and received by the demented persons.

#### Visiting the hospital for bio-medical care

Most of the key informants reported of ever taking the older persons for medical professional care at nearby health facilities. They further narrated that during the time no clear diagnosis was done on their patients (the older persons) as the health workers provided treatment for the prevailing signs and symptoms. The delay in definitive treatment could have given time for the infections to disseminate further to CNS region and cause further pathology including dementia. The narrative was typified by a participant by the saying below.

“*When the pain first started she kept visiting the hospital time and again to get medical help, she was given drugs without any thorough checkup to find the origin of the illness*” (participant 10, 57 years, male, Husband)

#### Mixing home-based care with hospital-based care

Some key informants narrated that their patients would get admitted in the hospitals but could not stay longer because of the treatment expenses they could not sustain admitted in the hospitals to receive the desired treatment and so gets discharged to receive further treatment from home. This prompted discharge of patients before full recovery from hospitals and receive the rest of care while at home without follow up from the health workers to assess improvements in the conditions of the patient.

“*She was discharged from the hospital after spending about three weeks, and advised to continue with her treatment from home. “The doctor said that my mother was much better but needed to continue taking some medication, and as said by the doctor, to-date my mother mostly suffers only from the effect on the brain, which causes her to lose memory quite often and fail to talk or make sense out of what people are saying, this is all that she is still going through once in a while”* (participant 3, 50 years, male, Husband)

#### Receiving herbal treatments

Some key informants also reported of their relatives (the older persons) sometimes used local herbs to treat themselves. They related this to the high financial constraints that their families could not raise to meet the cost of biomedical care. They also attached the condition of CNS infection and dementia to spirituality that did not require biomedical care and thus opted for spiritual care. This was exemplified by a narrative from a participant below.

*“The only treatment she received was local herbs that was given to her from the village and prayers that the church pastor came home and prayed when we told him of what was happening to her. We could not raise the money to pay for the drugs the doctors had prescribed for her”* (participant 11, 53 years, male, Husband)

#### Reducing pain with pain killers and antibiotics

Some key informants reported that their patients the older persons were being treated mostly with pain killers and antibiotics. Such presumptive treatment without specificity on the type of infection the medicines are targeted at instead escalated the condition. Participants reported that the presumptive treatment would palliate temporarily and then the patient’s condition reoccurs. This was exemplified by a narrative of a participant below.

*“She had only used the painkillers and antibiotics which helped her to reduce the pain. These drugs used to help her reduce the pain but was unfortunately just helping her temporarily and after sometime the pain gets triggered again and kept on increasing overtime”* (participant 2, 59 years, Sister)

### Theme 4: facing of a multitude of challenges to optimal diagnosis, treatment and care

We explored the challenges faced by the caregivers or family of the older persons with a positive history of CNS and probable or possible dementia while seeking the desired health care. This theme of Facing a Multitude of challenges to optimal diagnosis, treatment and care therefore consolidates all the diagnosis, treatment and care related challenges or difficulties encountered by the demented persons, their caregivers or family. The findings are described below.

#### Poverty delaying start of medications

Many of the participants expressed the lack of money was the main reason for the failure of their patients the older persons to receive the desired care. The lack of money for care could probably be due to limited the financial support for the elderly persons in the community. The lack of money hindered them from receiving quality care from quality health facilities which offered specialized care for CNS infections. The above narrative was typified by a participant in the saying below.

*“She faced financial constraints and couldn’t start medication. She spent a lot of money in the tests and because of this, purchasing the prescribed medication became a little challenging for her”* (participant 9, 55 years, female, Sister)

#### Getting improper diagnoses from the hospital

Some key informants also reported that the older persons lacked proper diagnosis in the initial stages of the infection. The inadequate or lack of diagnostic equipment’s in health facilities led to treatment of the presenting signs and symptoms without definitive understanding of the causative agents of the infection. This led to reoccurrence of the infection and long stays in hospitals. One participant narrated it as below.

“*When the pain first started she kept visiting the hospital time and again to get medical help, she was given drugs without any thorough checkup to find the origin of the illness*” (participant 10, 57 years. male, Husband)

#### Needing caregivers

Some key informants also reported that because of the forgetful nature of these patients, if not helped and reminded to take their drugs they end up missing taking the drugs in the right time. Therefore, their patients need a full time person to take care of them, since even their treatment take longer durations. One participant had the description below to narrate.

“*Old age is one problem I can say is affecting her memory because at this time she needs special attention they used to get but now they are not getting. The need someone to keep around and do for him most of the work, you can see he is even weak”* (participant 18, 62 years, female, married)

## Discussions

Our study explored the Central Nervous System (CNS) infection illness trajectory to dementia among the older persons with positive history of CNS infection illness and later life dementia in northern Uganda. The themes that emerged were four and they were the presentation of early symptoms of illnesses, manifestations of co-morbidities and or their medications, seeking and receiving of series of improper diagnosis, treatment and care and facing of a multitude of challenges to optimal diagnosis, treatment and care.

The early symptoms of CNS infection illness among older persons with dementia that emerged from interviews of key informants included chronic headache, neck pain, back pain and fatigue. These findings concur with the findings from a previous study conducted in the United States (US) where elderly patients with CNS infection illness presented with headache, fever, neck stiffness and altered mental status [30]. The implication of this finding is that older persons presenting with the aforementioned symptoms of illnesses would benefit from further diagnostic and screening tests to rule out neurological conditions including history of CNS infection illness and later life dementia.

Interestingly, our study found the existence of comorbidities of high blood pressure, CNS infection illness and dementia among older persons with positive history of CNS infection illness and later life dementia. There is limited literature on the comorbidity of high blood pressure, CNS infection illness and dementia. Whereas previous studies have shown separate associations between CNS infection illness and high blood pressure, high blood pressure and dementia, CNS infection illness and dementia [7, 14], the results of this study was based on subjective experiences and interpretation of the family caregivers, not an objective assessment and diagnosis approach. Therefore, epidemiological and mechanistic studies are needed to establish the epidemiological, cellular and molecular-level associations and pathways between high blood pressure and CNS infections among persons with probable or possible dementia.

Our study also found that some of the older persons with positive history of CNS infections and later life dementia were once victims of road traffic accidents involving traumatic injury to the head, neck or spine (brain and spinal cord) developed CNS infections later in life. The road traffic accidents involving traumatic injury to the head, neck, or spine have the potential to cause breakage in surface barriers such as the skin and bones allowing pathogens from the external environment to gain entry into the CNS and cause CNS infections. These findings agree with a previous study conducted in the US where CNS infection was also found to result from trauma and fractures involving the CNS [12]. These findings support the need to vaccinate victims of road traffic accidents against vaccine preventable CNS infections such as tetanus.

The treatment and care modalities received by the older persons with positive history of CNS infections and later life dementia in this study included a mix of biomedical care, home-based care, and local herbs. This finding concurs with findings from previous studies conducted in southwestern Uganda where caregivers or families of older persons with dementia were found to be seeking healthcare for their patient with dementia from both formal and informal care providers [31]. The similarity in the findings could due to the use of the same health policy in these two regions of Uganda which allows for plurality of healthcare providers including traditional complementary and alternative medicines providers. The association of neurological conditions including dementia to supernatural powers could explain the use of informal care seeking behaviors among the participants. The finding highlights the need for deliberate efforts that encourages care-related partnerships, collaborations and linkages between the formal and informal care providers to ensure older person received appropriate care they deserve for CNS conditions and dementia.

Our study also found that pain killer was among the treatment for pain management among the older persons with probable or possible and positive history of CNS infections. This could be as a result of pain being reported to be among the symptom of CNS infection the older persons with possible or probable dementia experience. This finding is in conformity with previous studies were analgesic were also reported among the medication for pain management among older patients with dementia [16, 17]. These findings call for the need to train health care providers including pharmacists and drug dispensers to do thorough pain assessment and investigations into the underlying causes of pain before prescribing or dispensing the pain killers to patients at high risk for CNS infections and dementia.

Our study in northern Uganda further found that older persons with history of CNS infection and dementia in later life used local herbs as one of the treatment and care modality for the neurological conditions. These results is in conformity with the previous studies were plant-based medicines were found to be the most frequently sought and used mode of treatment for a number of CNS disorders [18, 19]. The permission of a plurality of healthcare systems which includes traditional, complementary, and alternative medicines in Uganda and other Countries can explain the health-seeking behavior and the use of herbal medicines for the treatment of CNS conditions and dementia. However, more research is needed on the active ingredients in the local herbs used for the treatment of CNS infections among older persons with probable or possible dementia. The findings also call for the integration of traditional, complementary and alternative medicine providers into the mainstream formal healthcare sector by encouraging care-related partnerships, collaborations and linkages between them and the formal healthcare sector actors.

Our study found that poverty was one of the challenges hindering the older persons from obtaining timely and optimal health care for both the early CNS infections and the dementia in later life. As many of the caregivers made mention of their family’s inability to raise funds for carrying out diagnostic tests and procuring the drugs as prescribed by the healthcare professionals. This result concurs with the previous study conducted in South Africa where poverty and financial stress were highlighted as the most common barrier to providing care for older persons with dementia [20–22]. These findings point to the need for governments in Uganda and SSA at large to providing government funding to institutions or organizations that can extend free of charge health care services to the older persons with probable or possible dementia.

Our study in northern Uganda also revealed that patients with positive history of CNS infections and later life dementia encounter a series of missed diagnosis and mismanagements of their neurological conditions. This was probably due to the lack of equipped medical laboratories in most of the healthcare settings in northern Uganda being a post armed conflict area. These results conforms with findings in previous studies, were late diagnosis and syndromic approaches are used to treat CNS illness among older persons [32]. The findings underlines the need for training health care providers and equipping of health facilities to increase their capacities to conduct thorough laboratory investigations to confirm the exact neurological condition prior to initiating any form of treatment.

Lastly, our study in northern Uganda unearthed the need for government and partners to consider policies and mechanisms of care for older persons including those with the positive history of CNS infections and later life dementia to address their long course of treatment and care needs. The long time for treatment and care needs of the older persons with a positive history of CNS infections and later life dementia put their families not only in financial distress but also in emotional discomfort. Our findings conform to the findings of previous studies where caregivers were faced with similar challenges of a long course of treatment and extended hours of caring for the persons with dementia [20, 23]. These findings highlight the need for care providers to consider counseling services for caregivers and families of patients with a positive history of CNS infections and later life dementia. There is also a need for dedicated government funding for institutions or organizations providing institutional care for older persons including dementia cases.

## Study limitations

The current study in northern Uganda did not conduct laboratory investigations on the participants to confirm the positive history of the CNS infection illness. This was however addressed by obtaining additional data on the CNS infection illness from the past medical discharge forms of the participants. The natures of the CNS infection illnesses were written in the medical records as laboratory results or medical diagnosis for example ‘Cryptococcal meningitis’ although some of them were based on clinical history and physical examinations. Also, control for potential confounders for the association between CNS infection illness and dementia such as traumatic injuries were not possible in the qualitative research design but are well controlled for in the preceding quantitative study in the same setting which investigated the prevalence of CNS infections among older persons and its association with dementia among older persons in northern Uganda. Additionally, key informants who were caregivers of the index participants were the ones involved to provide data about the CNS infection trajectory to dementia of the index participants. The concurrent manifestations of multiplicity of symptoms in older persons made it not possible to examine what symptoms or comorbid conditions led to which type of treatment seeking. The reflexive thematic analysis of data as they manifest from participants resulted in multiple symptoms and factors with or without proven causative relations to possible or probable dementia.

## Conclusions and recommendations

Older persons with the positive history of CNS infection illness and later life dementia in rural northern Uganda were reported to present with symptoms of early life CNS infection illness ranging from neck pain, back pain, chronic headache, and fatigue. There were also reports of comorbidities particularly road traffic accidents involving traumatic injury to the head, neck, and spine, high blood pressure, chronic headache, and or their medications in the older person’s trajectory to later life dementia. A plurality of healthcare which included both formal and informal healthcare medicines were reported to be sought and utilized for the treatment and care of the CNS infection illness and the dementia without thorough checkup for the CNS infection illness. Sticking out were the reported challenges of poverty, and long course of treatment and care for the older persons with positive history of CNS infection illness and dementia which requires government interventions in the form of policies and mechanisms for funding of institutions or agencies to facilitate care for the older persons. Health professionals should conduct routine screening of older patients for history of CNS infection illness, chronic headache, high blood pressure, history of traumatic injuries to the head, neck and spine and their proper management to reduce the likelihood of dementia in later life. Further research with case–control or retrospective cohort design is recommended to characterize the trajectory of CNS infection illness to dementia including the time, duration and comorbidities among others.

## Abbreviations

ADRD: Alzheimer’s Disease and Related Dementia
BCSD: Brief Cognitive Score for Dementia
CHEWs: Community Health Extension Workers
CNS: Central Nervous System
HIV: Human Immunodeficiency Virus.
HSV-1: Herpes Simplex Virus – 1
RAs: Research Assistants.
SSA: Sub-Saharan Africa
US: United States
VHTs: Village Health Teams
WHO: World Health Organization

## Declarations

### Availability of data and materials

The datasets used and/or analyzed during the study are available from the corresponding author on reasonable request because the study is work in progress.
